# The pathology of embryo death caused by the male-killing *Spiroplasma *bacterium in *Drosophila nebulosa*

**DOI:** 10.1186/1741-7007-5-9

**Published:** 2007-03-15

**Authors:** Joanna K Bentley, Zoe Veneti, Joseph Heraty, Gregory DD Hurst

**Affiliations:** 1 Department of Biology, University College London, 4 Stephenson Way, London NW1 2HE, UK

## Abstract

**Background:**

Inherited bacteria that kill male offspring, male-killers, are known to be common in insects, but little is understood about the mechanisms used by male-killing bacteria to kill males. In this paper we describe the tempo and changes that occur during male-killing by *Spiroplasma *bacteria in the host *Drosophila nebulosa*.

**Results:**

*Spiroplasma *infected *D. nebulosa *males were developmentally retarded from 6–8 h into embryonic development at 25°C, and arrested at between stages 12 and 13 of embryogenesis (10–12 h). Dying males were characterized by a failure to form segments, and ultimately disintegration of the normal oval embryonic shape. Prior to death, dying males exhibited widespread apoptosis, as testified by TUNEL staining.

**Conclusion:**

The *Spiroplasma *kills male *Drosophila *in a narrow developmental period, shortly after the formation of the host dosage compensation complex that is required for male-killing. Male death is preceded by widespread apoptosis, but it is uncertain if this is primary or secondary apoptosis.

## Background

Male-killing bacteria, bacteria that are passed vertically from a female to both her male and female progeny and kill the males during embryogenesis, are known to occur in a wide variety of insect taxa [1]. Much research has been conducted on their frequency in natural populations, the identity of the bacteria concerned, their potential impact. This has revealed that they vary in frequency within and between host species, with prevalence varying from 1 to 99% of female hosts [2,3]. The bacteria are diverse, deriving from the genera *Spiroplasma, Wolbachia*, *Rickettsia*, *Arsenophonus*, and an unnamed representative in the Cytophaga-Flavobacteria group [4-8]. The impact on the host can be profound, with high male-killer prevalence associated with sex role reversal in the butterfly *Acraea encedon *[9], and the evolution of suppressor genes in *Hypolimnas bolina *[10].

In contrast, almost nothing is known about the timing or mechanism of male-killing with the exception of a few studies in the *Spiroplasma*-*Drosophila *system. Recent studies of this interaction have demonstrated that male-killing requires a functional host dosage compensation complex [11]. However, there is little precise knowledge regarding the timing of male death, and no information on the cellular pathology induced by the *Spiroplasma*. Past studies have described abnormalities occurring anywhere between cleavage divisions and 8 h after oviposition, with death occurring even in larval or pupal phase [12-14]. However, these previous studies are less than satisfactory because researchers were unable to identify the sex of embryos, so unhealthy embryos were automatically considered male. In addition, the focus of three of the four studies was an unnatural association: *D. melanogaster *transinfected with *Spiroplasma *from *D. nebulosa*. Whilst this transinfection does function with a good degree of penetrance, it is somewhat unstable in the laboratory, and these studies did not assess whether the infections functioned in the same way as a natural infection.

In this study, we examine the process of male death in a natural association, that between *Drosophila nebulosa *and its male-killing *Spiroplasma*. This strain of *Spiroplasma *is closely related to *S. poulsonii*, the male-killer discovered in *D. willistoni *[15]. This strain shows very high rates of natural vertical transmission, making the infection very stable and amenable to laboratory study. An antibody against SXL [16], the key switch peptide in *Drosophila *sex determination and present only in females, is used to allow the embryos to be sexed precisely. We first conducted a detailed study of the timing of male death caused by *S. poulsonii*, and the abnormalities observed. We also investigated the role of apoptosis in cellular death within male embryos at the point of male death. Previous work on bacteria-host interactions has demonstrated that bacteria are able to induce and repress apoptosis, both of which could result in the death of an embryo [17,17]. We tested whether apoptosis of host cells occurred during male death in the *Drosophila*-*Spiroplasma *system.

## Results

### Timing and morphology of male death

We first examined whetherthe SXL antibody raised from *D. melanogaster *SXL could be used to sex *D. nebulosa *embryos as it does in *D. melanogaster*. A comparison of the ratio of SXL-positive to SXL-negative *D. nebulosa *embryos in uninfected lines shows a 1:1 ratio of SXL+:SXL- embryos, as expected for a protein only present in females (see Table 1). Thus we conclude the antibody does allow differentiation of male from female *D. nebulosa*. There was no difference in the ratio of SXL-positive to SXL-negative *D. nebulosa *embryos between infected and uninfected lines (χ^2 ^= 0.0538, NS) indicating that this SXL expression is not altered by *S. poulsonii*, and can be used in infected as well as uninfected embryos.

**Figure 1:**
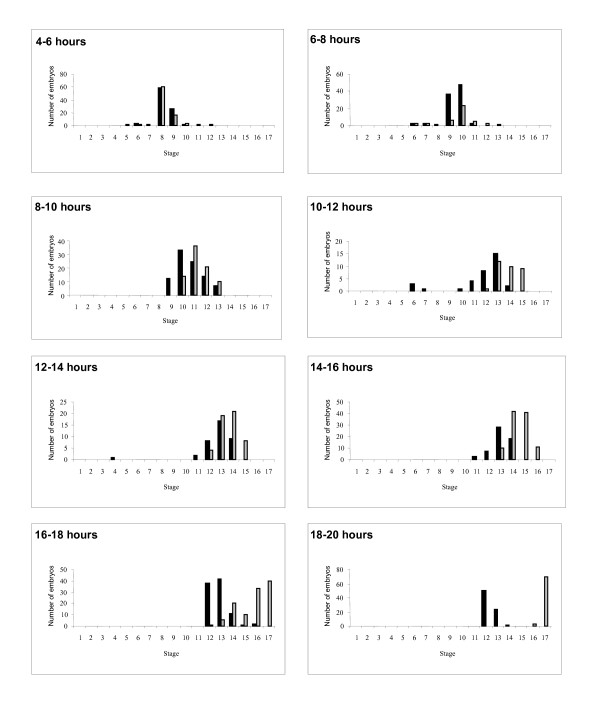
The developmental stage of male and female *D. nebulosa *embryos, infected with *S. poulsonii*, aged **4–20 **h AEL, in **2-h **cohorts. Black bars represent males, and grey bars represent females.

We then examined the timing of male embryo death by tracking the developmental stage attained by 2 h cohorts of eggs, infected and uninfected, allowed to develop between 4 and 24 h. Embryos in each cohort were placed into developmental stages delineated by Campos-Ortega and Hartenstein [18]. Infected males behind infected females in terms of development 6–8 h after egg laying (AEL), and by 10–12 h AEL there is a clear arrest of male development (Figure 1; data summarised in Table 2). Infected males arrested at or before stage 13 of development, whereas infected females continue through embryogenesis. Uninfected male and female embryos developed broadly in parallel, and at a rate equivalent to that observed for infected female embryos (Table 3).

**Figure 2:**
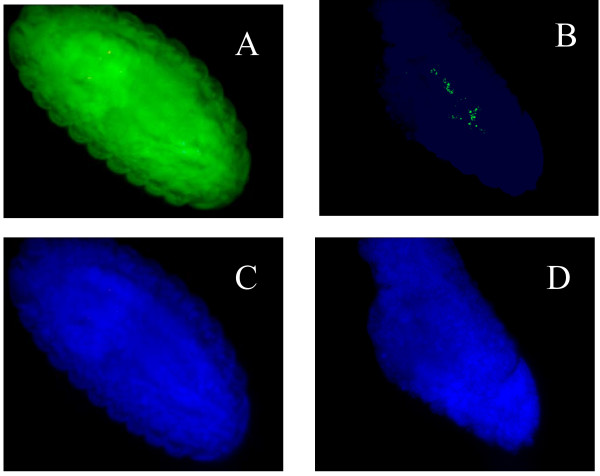
**Female embryo (left) and male embryo (right)**, both infected with *S. poulsonii ***(16–18 h **AEL). The top pictures, A and B, shows fluorescence with anti-SXL antibody in female and male respectively, and the bottom pictures, C and D, show the embryos structure under DAPI in female and male respectively. The female has developed normally and reached stage 16, the male has arrested before segmentation, and shows a characteristic amnioseral bulge.

The morphology of *Spiroplasma*-killed male embryos was very characteristic. Figure 2 shows a well-developed infected female embryo (stage 16, 16–18 h AEL) and an infected male of the same age. The only normal structure that is recognisable in the *Spiroplasma*-killed male is the cephalic furrow. There is an abnormal protruding 'sac' from the ventrum of the male embryo and the structure of the infected male becomes grainier and less regular from the point at which abnormalities are first noticeable. By this age, all nuclei in the embryo have broken down, and the embryo gives little hint of any internal structure under DAPI stain (Figure 2D). Male embryos arrest before segmentation (which is never observed), and around the point of germ band retraction.

### Are cells in male embryos dying by apoptosis?

**Figure 3:**
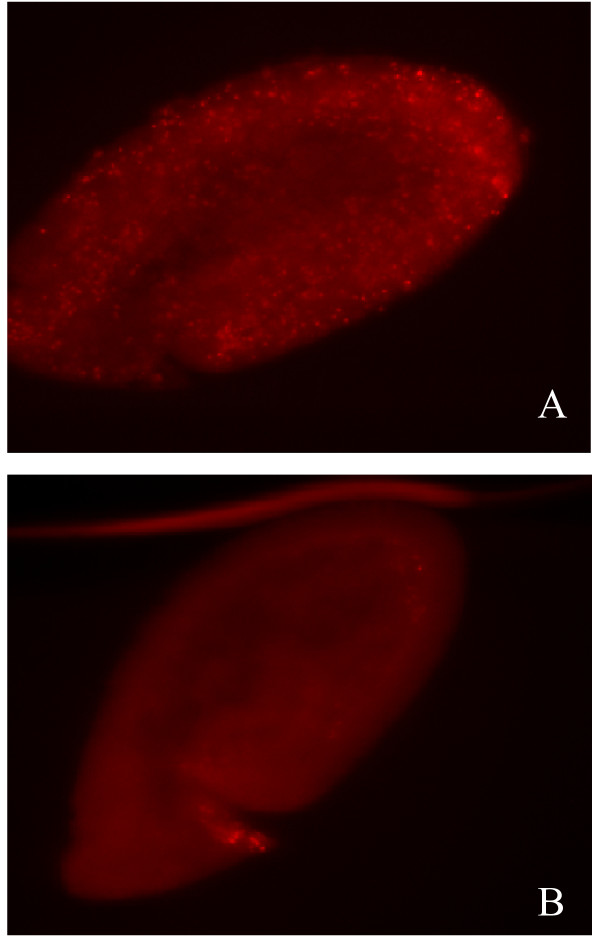
TUNEL staining of *Spiroplasma *infected male embryo (A) and female embryo (B), both at stage 10, **5–7 **h AEL. Red fluorescence indicates apoptotic nuclei; whilst the female embryo shows the characteristic pattern of apoptotic nuclei restricted to the cephalic furrow, apoptotic nuclei are observed throughout the male.

We examined the pattern of apoptosis in *Spiroplasma*-infected and uninfected embryos using the TUNEL procedure [19]. Infected *D. nebulosa *female embryos and uninfected embryos of both sexes were found to have apoptotic cells in the same pattern found in *D. melanogaster *[20], with TUNEL positive cells around the cephalic furrow, and sporadic TUNEL positive cells in the posterior of the embryo, from stage 10 onwards (about 7 h AEL). In contrast, infected male embryos of the same age show extensive apoptosis throughout the embryo, with vast numbers of TUNEL positive cells (Figure 3). The pattern observed in Figure 2 was seen in all male embryos derived from infected females (n > 200). It is notable that these infected male embryos become TUNEL positive before development completely arrests, as indicated by the widespread apoptosis in the stage 10/11 male embryo in Figure 3.

## Discussion and Conclusion

This paper investigated the development of male *D. nebulosa *embryos infected with male-killing *Spiroplasma*. It was observed that the infected male embryos become retarded from 6–8 h after being laid, and development was arrested completely at 10 h post egg-lay, between *Drosophila *embryo stages 11 and 13. No infected male embryo went beyond stage 13 of development. The infected male embryos have a very characteristic pattern of death, with failure to segment being observed at the point of germ band retraction, and eventually loss of the normal oval embryonic shape caused by the protrusion of an amniosera 'sac' through the ventrum of the embryo.

What was notable from this study is that the events involved with *Spiroplasma *induced male-killing proceed very rapidly. Over a 2-h period, relatively early in embryogenesis in absolute terms, the male embryos go from appearing phenotypically normal to showing widespread abnormality. The tight window over which this degradation occurs contrasts with previous studies, and probably reflects that male and female embryos could be discriminated precisely, allowing random deaths to be more precisely partitioned and a clear pattern observed. It may also reflect our study of a natural association where male-killing is being seen as a strong phenotype.

This study also examined the type of cell death occurring in dying males. It was observed that male-killed embryos were characterized by massive apoptotic death of cells within the embryo that preceded the complete arrest of development. Thus, we can conclude that the processes leading to male death are active at stage 10. It is notable also that male death occurs at the point at which natural apoptosis begins [20]. It is therefore tempting to hypothesize that male-killing operated through some subversion of the apoptosis machinery, inducing its uncoordinated expression of the apoptosis pathway across the embryo, that ultimately resulted in the arrest of development. However, it should be noted that the association of apoptosis with male death is not necessarily causal. There are many cases where apoptosis is induced by the host in response to infection rather than as a manipulation by the infection. In these cases of secondary apoptosis, death would have occurred with or without the apoptotic processes and cell death is merely a response to the pathology induced by the infection.

What remains totally unknown are the molecules secreted by the *Spiroplasma *that create the changes observed. At the most elementary level, we do not know whether there is a previously secreted factor that becomes active only at 6–10 h into male embryo development, or whether a factor is secreted *ab initio *in males only, from 6–10 h into development. Future work will need to focus on the nature of this factor and its interaction with host cellular and developmental systems.

## Methods

The male-killing infections used were from two *D. nebulosa *females (G37, G87) collected in Guadeloupe, during July 2001, previously described in Bentley et al. [15]. This infection was maintained in outbred *D. nebulosa *through mating of the females in each generation to males from an outbred population of *D. nebulosa *collected at the same time, and maintained at large population size. This population was additionally the source of the uninfected control population.

### Timing and morphology of male death

Large groups (>100 individuals) of *Spiroplasma *infected line G37, *Spiroplasma *infected line G87, and uninfected *D. nebulosa *aged between 2–14 days old, were placed onto separate grape juice agar laying plates. Following a prelay to avoid oviposition of retained embryos, the flies were transferred onto new plates for 2 h. In order to obtain a time series of embryos, the eggs from the 2-h collections were then aged for the required time (between 4–22 h) at 25°C. This was conducted for each of the outbred stocks of the *S. poulsonii *infected lines and the uninfected outbred stock. The flies were aged to produce near-complete vertical transmission efficiency, which is observed in this stock under these conditions.

Following development, embryos were collected and fixed by standard methods as described in Sullivan et al. [21]. In brief, the eggs were dechorionated by shaking in 3–5% bleach followed by washing in PBS. They were then fixed through shaking in a 1:1 mix of heptane and 4% paraformaldehyde in PBS, and devitellinized through shaking in an equal 1:1 mix of 90% Methanol/10% EGTA and heptane. Devitellinized embryos (those that had sunk to the bottom of the methanol phase) were then collected and stored in Methanol. Fixed embryos were rehydrated into PBS bearing 0.1% triton X100 (PBT) and then stained with two antibodies; mouse anti-SXL (Developmental Studies Hybridoma Bank, University of Iowa) and rabbit anti-MLE (courtesy of Prof. M. Kuroda, Howard Hughes Medical Institute, Madison USA). Anti-SXL binds SXL, is female specific, and visible 1.5 h into embryonic development in females only [16]. Anti-MLE is used as a positive control for antibody staining, visible in both males and females [22] where the fixing and staining procedure has occurred corresecondary ctly. Unbound antibody was removed by washing in PBT, and 1 μl of FITC-conjugated rabbit anti-mouse IgG secondary antibody, and TRITC-conjugated goat anti-rabbit IgG (both Molecular probes) added, followed by washing. Unbound secondary antibody was removed by washing in PBT. Embryos of a given age and infection status were mounted together on a slide in Vectashield containing DAPI, and visualised on a fluorescence microscope.

On slides where embryos were positive for MLE binding, embryos were scored for sex using SXL fluorescence (FITC positive = female), and developmental stage using DAPI staining to reveal structure. Developmental stage was categorised as per Campos-Ortega and Hartenstein's description of *D. melanogaster *development that segregates embryogenesis into 17 stages [18], from newly fertilized zygote through to formed larva. Note that not all embryos were scored: inequalities sex ratio in the observations are associated with differential scoring of embryos by sex in certain cases, rather than biological differences.

### Are cells in male embryos dying via apoptosis?

The TUNEL procedure was used to label the 180 bp DNA fragments found in apoptotic cells and make them visible under a fluorescence microscope. In *D. melanogaster*, apoptosis is seen from stage 11 onwards (7 h AEL), in a dynamic yet characteristic pattern within the embryo [20]. With this is mind *D. nebulosa *embryos were tested in cohorts 3–5 h, 5–7 h, and 7–9 h AEL for the presence of apoptosis in either Spiroplasma infected or uninfected embryos. Embryos were also stained with anti-SXL as previously to identify their sex. Embryos were collected and fixed as above and the *In Situ *Cell Death Detection Kit, TMR red (Roche) was used to detect apoptosis. The protocol was adapted slightly from the manufacturer's instructions for detecting apoptosis in cell suspension material. The embryos were washed twice in PBS and then the PBS was removed and 50 μl of the TUNEL reaction mixture was added. The embryos in TUNEL reaction mixture were left for 1 h at 37°C in the dark before being washed three times for five minutes in PBS. Embryos were then stained for anti-SXL and mounted and examined as described above.

During this process, a negative control was also completed as per the manufacturer's instructions. No positive control was necessary, as from 7 h onwards *D. melanogaster *embryos naturally have some apoptosis visible [20], and this paper has already demonstrated that *D. nebulosa *embryos should also have apoptotic cells naturally occurring in that timeframe (as they will have also reached stage 11). When TUNEL staining was being performed on embryos younger than 7–9 h AEL an older embryo sample was subjected to the test at the same time to act as a positive control.

## Authors' contributions

JB and GH conceived the project. JB and JH conducted the observations of male death pattern, with advice from ZV and GH. JB conducted the TUNEL assays with advice from ZV and GH. JB, ZV, JH and GH wrote the paper.

## Figures and Tables

**Table 1:** The number of individual embryos staining negative (SXL^-^) and positive (SXL^+^) for the presence of the protein SXL in batches of eggs laid by females uninfected and infected with *S. poulsonii*, allowed to develop between 4-24 h AEL.

	Infected	Uninfected
SXL^-^	752	307
SXL^+^	714	298

**Table 2:** Median stage of development of male and female *S. poulsonii *infected *D. nebulosa *embryos of ages 4–24 h AEL. () = number of embryos. Wilcoxon test results compare male and female development stage.

**Cohort (h AEL)**	**Median stage of embryonic development**		**Wilcoxon test results**	**Significance**
			
	**Male**	**Female**		
4–6	8 (90)	8 (85)	8025	0.705
6–8	10 (93)	10 (41)	5549	0.0062
8–10	10 (91)	11 (81)	6617	0.0001
10–12	12 (34)	14 (32)	740	0.0001
12–14	13 (37)	14 (52)	1213	0.0005
14–16	13 (56)	14 (104)	2294	0.0001
16–18	13 (92)	16 (100)	5120	0.0001
18–20	12 (75)	17 (72)	2850	0.0001
20–22	12 (170)	17 (145)	4196	0.0001
22–24	13 (14)	17 (2)	105	0.0005

**Table 3:** Median stage of development of male and female *S. poulsonii *uninfected *D. nebulosa *embryos of ages 4–24 h AEL. () = number of embryos. Wilcoxon test results compare male and female development stage.

**Cohort (h AEL)**	**Median stage of embryonic development**		**Wilcoxon test results**	**Significance**
			
	Male	**Female**		
4–6	8 (39)	8 (40)	1531	0.749
6–8	10 (42)	10 (35)	1688	0.581
8–10	11 (82)	11 (80)	6356	0.243
10–12	11 (13)	12 (13)	170	0.761
12–14	14 (39)	13 (39)	1642	0.282
14–16	13.5 (40)	14 (34)	1291	0.0264
16–18	14 (25)	15 (19)	40.5	0.0001
18–20	17 (21)	17 (28)	5075	0.4083
20–22	17 (2)	17 (5)	N/A	-
22–24	17 (4)	17 (5)	N/A	-
